# Refugee and Immigrant Community Health Champions: a Qualitative Study of Perceived Barriers to Service Access and Utilisation of the National Health Service (NHS) in the West Midlands, UK

**DOI:** 10.1007/s10903-021-01233-4

**Published:** 2021-06-18

**Authors:** Oliver Mudyarabikwa, Krishna Regmi, Sinead Ouillon, Raymond Simmonds

**Affiliations:** 1 Department of Public Health, Coventry University, Coventry, UK; 2 Institute for Health Research, University of Bedfordshire, Luton, UK; 3 Centre for Trust, Peace and Social Research, Coventry University, Coventry, UK; 4 MiFriendly Cities Refugee and Migrant Centre, Coventry, UK

**Keywords:** Refugees and immigrants, Community health champions, Primary care, Barriers, Utilisation, NHS

## Abstract

There has been much discussion recently that better healthcare systems lead to increased service access and utilisation. However, there are still concerns raised among the refugee and immigrant communities about barriers to access and utilisation of primary healthcare services in the UK. This study aimed to explore with refugee and immigrant community health champions (CHCs) their perceptions about such barriers based on feedback in their own discussions with fellow refugees, asylum-seekers and immigrants in the West Midlands, UK. A total of 42 refugees and immigrants were recruited. Qualitative design-focused group discussions were conducted among purposively selected participants. These discussions were conducted between May and September 2019, and data were analysed using thematic analysis. The barriers to service access and utilisation are categorised into four themes: (i) knowledge about health issues that most affected refugees and immigrants; (ii) community indications of factors that obstructed service access; (iii) challenges in identifying local teams involved in service provision; and (iv) accurate knowledge about the different teams and their roles in facilitating access. This study higlighted that the levels of service access and utilisation would depend on the competence and effectiveness of the health system. Urgency and seriousness of individuals’ healthcare needs were the factors that were perceived to strongly influence refugees and immigrants to seek and utilise local services. We identified a number of potential barriers and challenges to service access and utilisation that should be overcome if primary healthcare service is to be planned and delivered effectively, efficiently and equitably in the West Midlands.

## Introduction

There has been much discussion recently that better healthcare systems lead to increased service access and utilisation, and commitment to safeguard the health and wellbeing of more vulnerable people [1]. There is also realisation that service-users not only have increased expectations for quality services but also demand information about how to improve their healthcare experiences [2]. These developments are the results of having better knowledge about their health needs and effective ways of satisfying them. Objectives of most governmental healthcare organisations are therefore to improve citizens’ experiences in accessing quality, safe and affordable healthcare and services [3, 4]. Two important principles guide their healthcare activities. First, there is the interest in achieving equity in employment of national resources in ways that help disadvantaged sections of the population to benefit by improving their health [5, 6]. The second principle concerns the desire for healthcare providers to prioritise efficiency and effectiveness in their activities to improve population health [7]. These factors drive healthcare providers to influence their communities to become more proactive in the utilisation of available and essential primary care services.

Global healthcare principles and objectives reflect consensus that health problems affecting people are due to avoidable injustices and unfairness within national health systems [8]. With regard to refugees and asylum-seekers, for example, legislation for mandatory testing for infectious diseases may exacerbate health inequalities by driving away some people from the service if their health status determines their settlement in the country [9]. Some of the inequalities are influenced by structural flaws in arrangements for service delivery. For example, Smith [10] argues that some health systems tend to experience wide disparities in community health outcomes because they are not designed to cope with increased refugee and immigrant populations. Such healthcare systems may be missing out on potential benefits like contributing to efficiency if skilled refugees and immigrants are employed as cheaper talent and expertise to meet their own communities’ health needs [11].

Within the UK, community-centred models of health service delivery have been used due to a tradition of encouraging local-level providers to evaluate inequalities in service utilisation in order to inform policies which promote equitable access [7, 11]. More recently, health leaders have reaffirmed preparedness to achieve universal health coverage by engaging refugee and immigrant community health champions (CHCs) to use their experience and knowledge about local health problems to influence higher service utilisation by individuals within their refugee and migrant communities [12].

Particularly in the UK, local health authorities prefer the CHCs initiative for its likely potential to improve a range of health indicators through influencing vulnerable communities to increase their utilisation of primary care services [13]. Policies that encourage non-discriminatory access to publicly financed healthcare services are organised around GPs and walk-in centres as the entry points that are complemented by different types of charities and third sector agents [10, 11]. In the West Midlands (UK), for instance, significant refugee, asylum-seeker and immigrant populations are particularly relevant to suboptimal service access and utilisation [14]. A fear frequently cited is that service providers may be government informers on people without legal settlement [14].

Many European countries have raised concerns about influx of refugees and immigrants contributing to national inequalities in health [9, 10]. Access to healthcare by the new arrivals is often constrained by the lack of information and restrictive policies. Most countries have therefore been challenged to promote universal healthcare access, by adopting principles for providing the new arrivals with information about essential services and how they may be accessed and utilised [8, 9].

In the UK, the National Health Service (NHS) adopted flexible opening hours, and continues to offer free services plus patient access to a range of teams who have expertise in specific health issues [11, 15]. Yet Public Health England [12] reckons that poor health outcomes of refugees in places like the West Midlands are explained by lack of propensity to utilise services nearer their homes. The easiest reasons to blame are their misconceptions about barriers to access [13], poor appreciation of factors that negatively affect their health [11], and lack of relevant advice on how they can get help from health teams within their communities [6]. However, limited evidence exists in relation to what the refugee and immigrant populations perceive to be significant factors that obstruct access to and utilisation of services in their areas. This study aimed to explore, with refugee and immigrant CHCs, their interpretations of the barriers to service access and utilisation emerging from their discussions with fellow refugees, asylum-seekers and immigrants in the West Midlands, UK.

## Methods

### Study Design

A qualitative study was conducted in the UK from May to September 2019. The primary data came through refugee and immigrant CHCs using familiarity with their own communities to explore their colleagues’ perceptions on barriers towards access and utilisation of locally provided healthcare services. Focus group discussions (FGDs), involving the CHCs as participants, were then employed not only to explore individual interpretations of their colleagues’ indications about the service access and utilisation barriers but also to understand people’s cultures, customs, habits and mutual differences [16, 17]. In fact, a qualitative study is a social inquiry strategy that usually emphasises meanings of words rather than quantification of data to explain people’s experiences of a phenomenon [16, 18]. This inquiry was conducted in the metropolises of Birmingham, Coventry and Wolverhampton located in the West Midlands.

### Sampling

We used non-probability purposive sampling [16] of refugee and immigrant CHCs (n = 42) enrolled for public health training under the Migrant Friendly Cities project, to be the primary data collectors. Individual CHCs were given homework to independently engage with fellow refugees and immigrants using any convenient and helpful methods such as informal individual or group discussions to get their indications of healthcare issues that affect them and the barriers to service access and utilisation. Using such approaches would require the CHCs to conveniently determine the sample sizes of informants on the basis of theoretical saturation [18, 19].

### Data Providers

Data were provided at two levels. The ordinate data providers were people who identified themselves to the CHCs as either refugees, asylum-seekers or immigrants. A recent census report [20] indicated a fairly high population density of refugees and immigrants across the metropolises. It convinced us that the population concentration would give us sufficient data to better understand possible health issues and barriers to access without stipulating or fixing the number of informants to be reached by the refugee and immigrant CHCs [18].

The refugee and immigrant CHCs were the subordinate data providers through reporting and participating in class discussions, which we treated as a form of FGDs. They were a non-homogenous group of people (see Table 1) who originated from 22 countries spread across Africa, Asia, Europe, and South America. The social and demographic statuses did not hinder their ability to give feedback on collected data, or to interpret the health issues and barriers to service access as reported by their fellow refugees and immigrants.

**Table 1:** Socio-demographic characteristics of respondents

Total (n = 42)	Completed: n (%)
Characteristics	42 (100)
Sex	
Male	15
Female	27
Age group	
16—28	2
29—39	31
40 ≤ 65	9
Highest education completed	
Secondary school	10
Further / higher education	32
Professional qualification	
Nursing	5
Teaching	5
Others	11
Without professional qualification	21
Employment status	
Full-time work	12
Not working	18
Volunteering	12

We adopted the International Office of Migration’s [8] definition of refugees and immigrants to determine participants in the public health training course, and thus, the data providers for this analysis. This population group are people forced to flee their home countries by different kinds of circumstances which constitute dangers to their lives [8]. In this analysis we included people who find it hard and unsafe to return home if the forces that pushed them out continue to exist, while their status as refugee was yet to be legally determined by the government. The ordinate and subordinate data providers therefore needed to have been in the UK for a minimum of six months to have basic knowledge about the process of seeking healthcare, including registering with a local GP [13, 14, 21].

### Data Collection

The methods included ordinary discussions with individuals or groups, face-to-face and telephonic interviews, and colleague observation, culminating in FGDs in classroom settings. Data were collected between May and October 2019. Two phases of data collection were used.

The first phase concerning CHC data collection from fellow refugees and immigrants was essentially an open inquiry. It discouraged us from pre-determining a minimum number of occasions or the number of informants to be reached by the data collectors, though they eventually reported reaching the range of 2–8 informants per occasion with discussions lasting approximately 55–75 min. The second phase concerned the data collectors giving feedback in classroom settings culminating in FGDs facilitated by the researchers. FGDs can be considered not only as an efficient approach “to capture the perspectives of multiple participants on a single occasion”, but also “offer an additional method for triangulation” [22], p.129].

Initially, 44 CHCs of refugee and immigrant backgrounds were enrolled for public health training between the three metropolises, but the drop-out rate before field assignment was 4.5% (n = *2*). All the remaining CHCs (n = *42*) had three (n = *3*) opportunities to contribute by collecting field data. Their first assignment upon induction was to investigate health issues that affect refugee and immigrant people within their areas. The second and third field tasks (weeks 2 & 3) required them respectively to investigate barriers to service access and utilisation; and the population’s knowledge about the range of healthcare agents, charities, or governmental teams that can impact their health through offering help on health and related social care. Provided they all complied, this would provide 126 sets of data deriving from topic guides [23] focusing on two important areas: (a) common health issues affecting individual refugees and immigrants, and (b) barriers to access including knowledge about the range of helpful service providers. The tally of informants would be indeterminable in light of fluidity of the inquiry and data collection methods. The data collection period coincided with Black History Month, which offered the CHCs an opportunity to reach more informants to complement contributions by congregants at church services and social clubs. The topic guide was piloted with seven CHCs to improve validity and relevance of the questions [24, 25].

We considered a classroom discussion with CHCs to represent an FGD to unpack the issues in their individual interpretations of barriers to healthcare access. Nine (n = *12)* FGDs were eventually held between the metropolises over six months. The average time per FGD was 120 min and all were facilitated by the researchers, and contributions restricted to refugee and immigrant CHCs.

### Data Analysis

Data were analysed using a thematic analysis approach. As Bryman [20] suggests, we search for themes from the field data, and from the themes which emerge, we then develop codes that relate to the research focus. Ryan and Bernard’s [26] approach was undertaken to develop coding using these steps: (a) repetition—pay more attention to the topics that occur again and again in the transcripts, (b) indigenous typologies or categories—local expressions that are unfamiliar or used in an unfamiliar way, (c) metaphor and analogue—ways in which participants represent their thoughts in terms of metaphors and analogues, (d) similarities and differences—how participants discuss or explore a topic in different ways, (e) missing data—reflecting what is not in the field data, and (f) theory-related materials—using scientific concepts or ideas as a springboard for themes.

## Results

The results indicate the views of: (i) individual refugees and immigrants who participated in FGDs that were facilitated by CHCs with refugee and immigrant backgrounds, and (ii) CHCs’ interpretations of what their colleagues believed to be barriers to their service access and utilisation.

### Demographic Profile

In this study, we focus on summative data that were contributed by the refugee and immigrant CHCs since they were the only ones we had direct contact with through our facilitation of the FGDs in classroom settings. They were all (n = *42*) assigned homework to investigate health problems and levels of service utilisation in their communities as part of their training on public health practice. Across the metropolises, CHCs were a heterogenenous group of data collectors aged between 29 and 65 years (n = *32*) (75%) and mostly females (64.3%). The majority (76.21%) had higher education qualifications to degree level (Table 1), making them capable of collecting basic data as CHCs are expected to.

These socio-demographic characteristics, and the fact that the CHCs originated from countries across the world, to an extent influenced their different experiences in local areas, and challenges experienced in interpreting to synthesise community features, risky lifestyles by individuals, and knowledge about hotspot areas for health problems that contributed to possible barriers to proactive health improvement by increasing local healthcare service utilisation.

Using thematic analysis, the findings from this qualitative study were categorised into five interlinked themes:

- Perceived health problems;
- Barriers to access and utilisation;
- Predisposing factors; and
- Priority causes of illness and deaths.

### Perceived Health Problems Affecting Refugees and Immigrants

After adjusting for marginal variations in lifestyles and community differences, mental health illnesses, physical incapacitation, drugs and alcohol abuse, modern slavery, sexual abuse and infectious disease exposure emerged as the significant problems impacting on the health and wellbeing of refugees, asylum-seekers and immigrants. Across the three cities, those who had settled for longer than 10 years had better knowledge about NHS arrangements for care access, which made them more likely to utilise healthcare services. While significantly less affected by health problems compared to recent arrivals (< 5 years), their level of service utilisation was to an extent still suboptimal.

Health problems concerning increased drug and alcohol abuse, and exposure to infectious diseases were identified. Both the male and female individuals were affected as they tend to use substances as solace for missing home, families and friends*.* To an extent, *“some of them become careless and risk poor health and sometimes death, by abusing drugs and alcohol to treat their loneliness”.* Desperation deployed by refugees and asylum-seekers was also found to increase their vulnerability. It drove some into modern slavery, which further risked their physical health through overworking, sexual exploitation and serious infectious diseases including HIV/AIDS (Table 2).

**Table 2:** Perceived major health issues affecting refugees and immigrants

Mental health problems	Modern slavery
Physical health and incapacitation	Sexual abuse and
Drug, substances & alcohol abuse	Infectious diseases – STI, HIV/AIDS & tuberculosis

Meanwhile, mental health illnesses and sexual abuse were found to be significant health problems. However, those from Africa and Asia *“do not openly discuss mental health illnesses and sexual abuse for reasons to do with cultural beliefs or feeling ashamed*”. The people who experienced these problems were associated with mainly drug, substance and alcohol abuse. Those with psychological problems were mostly previous victims of some forms of torture in their home countries. They were still haunted by their experiences *“(…) of escaping from home, and being denied food, shelter and treatment of their injuries”* so many years after arriving in the UK. The experiences drove them into desperation to accept suboptimal conditions, even sharing *“private accommodation that have neither furniture nor heating with strangers”* compared to returning home*.* Yet their status as refugees and immigrants frightens them from complaining and continue to experience unconducive conditions.

### Barriers to Access and Utilisation

We analysed the data to understand some of the possible barriers to refugees, asylum-seekers and immigrants taking actions to avoid these health risks. What emerged were ideas around themes described in Table 3. Most refugees and immigrants in the UK are so convinced that they benefit from continuously searching for more comfortable places for settlement. Being transient was, however, perceived to be a behavioural and systemic barrier to getting continuous care by health professionals. This is not because they initiate the movements themselves. On the contrary, the government often settles successful applicants for refugee status in regions other than the West Midlands where the initial application was made. By frequently changing their residential locations and cities, immigrants generally perceive it as non-prioritisation of their individual health and wellbeing. It also prevented them from maintaining their homes in order to live in conducive environments.

**Table 3:** Possible explanations for experiencing health risks

Transient nature of refugees and immigrants
Neglecting home upkeep
Not prioritising own health
Unsettled lifestyles and individual behaviours
Inability to pay for healthcare
Fear of being reported and deportation by the authorities

Refugees, asylum-seekers and immigrants were described as depicting chaotic behaviours and unsettled lifestyles, which were perceived to be barriers to disease avoidance and healthcare service utilisation*.* There was concern that *“it didn’t help care professionals to understand effectiveness of their activities if people are “sofa-surfing” and wilfully not complying with getting treatment and rehabilitation services”.* This is not because they would have recovered. Most of them will still be sick but cannot be easily traced by the professionals because they lack permanent addresses.

Inability to pay and lacking the right papers to register with GPs also emerged as two important barriers to access. Participants in the FGDs believed that *“presenting to GPs with all the paperwork which they ask for was risky”.* There is a mythical fear of possible deportation if their information was passed on to the authorities. These misconceptions prevented especially undocumented refugees and asylum-seekers from registering to utilise primary care services, even for serious illnesses.

### Predisposing Factors

The principal factors for experiencing prominent health problems across the metropolises were perceived to hinge on circumstances forcing one to become a refugee or immigrant, and issues in getting settlement documents. Those who fled torture or extreme cases of discrimination in their home countries were found to distance themselves from social activities. Despite robust protections for lesbian, gay, bisexual and transgender (LGBT) rights in the UK, refugees of such sexual orientations, especially those from Asian and African countries feel their *“compatriots are not progressive and remain very homophobic like those back home”.* These factors prevented affected people from getting the benefits of possible rehabilitation and universal care provided by the NHS. Reliving some heart-wrenching episodes of previous lives also usurped their confidence to seek help from relevant professionals.

The analysis also revealed that increasing numbers of young and adult male and female refugee and immigrant people across the board were taking up drinking and tobacco-smoking habits. A male CHC was surprised that his *“(…) barber from Africa had “customers” who regularly used the shop as a place for drinking and listening to music from home”.* The health consequences of their habit are disregarded, even though an increased number of males in that particular community group is showing evidence of social problems involving alcohol, drugs and substance abuse.

Having children was also identified as an important predisposing factor with mixed effects on refugee and immigrant people: (i) experiencing health problems, and (ii) propensity to utilise available services. Regardless of circumstances leading to their current status, refugees who left children and family dependents tended to take illegal and risky jobs. Those with permission to work *“take multiple jobs, worked long hours and postpone seeking healthcare because they want to continue earning”* for their subsistence and remittances home.

The refugees and migrants in this study originated from across the world. Their individual behaviours, culture and lifestyle choices therefore differed in their predisposition to risks of diseases. While the participants identified increased frequency and levels of drinking and tobacco-smoking among refugee and immigrant people, it affected those from African countries south of the Sahara and the Caribbean more than other regions. It contributed to high prevalence of diabetes, heart disease, stroke and cancer in refugee and immigrant people of African and Afro-Caribbean backgrounds.

### Priority Causes of Illness and Deaths

A comparison of priority health issues in immigrant communities against officials’ indications about the major public health problems (Table 4) revealed mixed perceptions. Participants in the FGDs were explicit about drug, substance and alcohol abuse-related illnesses as problems affecting their communities. They only also inferred stroke and lung cancer as significant problems due to their respective causal relationships with “physical incapacitation” and “increased rates of tobacco-smoking” by people in their communities.

**Table 4:** Indications of top-six causes of illnesses and deaths in the West Midlands, UK

Infant mortality	Alcohol-related illness
Coronary heart diseases	Lung cancer
Respiratory infections	Stroke

Meanwhile, public health officials identified infant mortality, heart disease, and acute respiratory infections as major problems across the metropolises. With regard to infant mortality in refugee and immigrant communities, participants in FGDs associated the problem with government disrupting continuity of antenatal care for pregnant women by moving them between locations. It increases infant mortality, especially since the women already experience other forms of barriers to service access linked to refugees and immigrants. Community-based investigations by this study also found infectious disease, sexual abuse and modern slavery as much more problematic in the West Midlands.

## Discussion

Our study showed the important role of factors concerning NHS structures and competence, and immigrant community characteristics and uncertainty about their status in influencing service access and utilisation. It showed that unpredictability in the number of immigrants arriving in the country and where they will choose to settle will always make health providers uncertain about what services to prioritise [9, 10]. By the time health problems surface in one city, their prevalence will be high in many others [11]. High levels of service access and utilisation depend on competence and effectiveness of the health system to facilitate better understanding of health priorities at community and individual levels [27, 28]. There is a gap between NHS priorities and refugees’ and immigrants’ knowledge about how to access the services. Closing the knowledge gap is achievable provided the NHS continually explores understanding local-level healthcare priorities using models such as the CHCs endorsed in the West Midlands. This analysis showed that the CHCs model can be helpful in creating awareness about healthcare problems and ways to upscale utilisation by refugees and immigrants without infringing the rights.

The analysis revealed that refugees’ and immigrants’ indications about barriers to access are questioned for reliability [8]. This may be a sign for the NHS to take caution in formulating strategies for universal healthcare coverage. Immigrants may be misinterpreting the barriers due to inconsistency in guidance for service access. In the NHS, too many agents with interests in refugee and immigrant health and wellbeing may be issuing conflicting guidance. Thus universal healthcare access can be achieved provided refugee and immigrant people are certain and honest about their experience in seeking care. Being candid about the barriers can be worth a lot more than judgements of local health agents who may issue misleading information. There is no evidence of the NHS leadership confusing service-users by issuing mixed messages that limited their ability to improve health by utilising available services [2].

An important implication of this analysis concerns the potential contribution of CHCs in achieving universal health coverage. Interventions towards this objective are hard to design, especially without all the correct facts about health problems and perceived obstacles to service utilisation [8]. One of the major contributions of this analysis was to highlight the advantage of recruiting refugee and immigrant CHCs to investigate health issues in their own communities. It is a feasible strategy for understanding pertinent information on this group of people’s health-seeking behaviour and the barriers they face [13, 14]. This study also contributed to highlight that much about what refugees and immigrants perceive to be their major health problems and why they may fail to seek help by experts is either not clearly understood, or is sometimes at variance with official opinion.

These results may explain why studies in some developed countries consistently advocate self-reporting of health problems [2, 27, 29]. In this analysis it proved to be a helpful and sensitive indicator of healthcare services for prioritisation at local levels within the NHS. There is a feeling of empowerment when people who directly experience health problems get the chance to openly share their pertinent reasons for not utilising available services [5]. More importantly, professionals benefit by using the knowledge to design better informed interventions because their informants are familiar with socioeconomic circumstances that obstruct patient access [29]. The data central to this analysis were contributed by people who are not homogenous in many respects. Inevitably, awareness about helpful mechanisms to access essential healthcare services differed between participants in FGDs. However, it is found that this had minimal impact on efficacy if participant contributions were correctly evaluated to understand what may help individuals to be proactive to improve their health [1, 3, 30].

The West Midlands, where this analysis was based, is a relatively poor region associated with low cost of living [11]. It attracts refugees and immigrants to settle there for a number of benefits, including saving money for remittance to home countries [9]. The consequences include the cities experiencing challenges in dealing with new and diverse cultures, lifestyles, and individual circumstances which impact residents’ health indicators [20, 31]. This analysis sheds light on some of the important reasons why service access and utilisation by refugees and immigrants may be genuinely low. The most likely reason could be that frugal service providers are tempted to restrict access for irregular immigrants [9, 27], especially if they feel their facilities, originally designed for small population sizes, are now overwhelmed by a growing population with different healthcare needs and expectations [15].

These findings also chime with the argument that there is correlation between, on the one hand: (i) people’s perceptions about their health status, and (ii) ability to pay; and their decisions to utilise the services on the other [2, 6]. Immigrants across the region obviously utilise more hospital clinical services than those provided at GP surgeries. It makes us conclude that, if people consider their health conditions as life-threatening, they tend to seek help. Conversely, if they think their conditions “self-limiting”, they are unlikely to seek care [32]. Also, even where especially refugees and asylum-seekers are certain about the seriousness of a health condition, the drive to seek help diminishes if they are expected to pay [30].

Across the UK, these relationships and some of the perceived barriers largely reflect refugee and immigrant people’s myths and misconceptions about accessing healthcare. There is evidence of government’s generous facilitation of universal healthcare through issuing guidance that clarifies immigrants’ health rights [13]. This is in addition to the NHS extending free treatment of infectious diseases, maternity care, and mental health illness to everyone, including undocumented immigrants, based on need rather than ability to pay [7, 13].

## Limitations

The study had a number of limitations. First, it was primarily focused on the barriers to service access and utilisation from the perspectives of only refugees and immigrants. Focus on this specific population group gives the study a relatively small sample size compared to investigating other citizens. Second, the analysis is limited to only three metropolises in only one region of the UK. Third, all the data were collected using only FGDs, and a quantitative or mixed methods study would have provided a better, more representative view of the informants.

## Conclusion

This study highlighted a number of factors or issues at community and individual level, health delivery level, and contextual characteristics which predispose refugees and immigrants to diseases and limited utilisation of services. These might be helpful checklists of potential barriers to access to and utilisation of local primary care healthcare services to refugees, asylum-seekers and immigrants. Further research is needed to find the best methods of addressing these issues so that primary care health services at the local level could be planned and delivered effectively, efficiently and equitably.
